# LB2307. Safety and Efficacy of Letermovir (LET) versus Valganciclovir (VGCV) for Prevention of Cytomegalovirus (CMV) Disease in Kidney Transplant Recipients (KTRs): A Phase 3 Randomized Study

**DOI:** 10.1093/ofid/ofac492.1897

**Published:** 2022-12-15

**Authors:** Ajit P Limaye, Klemens Budde, Atul Humar, Julia Garcia-Diaz, Robert P Carroll, Yoshihiko Murata, Valerie L Teal, Christopher L Gilbert, Barbara A Haber

**Affiliations:** University of Washington Medical Center, Seattle, WA; Charité-Universitätsmedizin Berlin, Berlin, Berlin, Germany; University Health Network, Toronto, Ontario, Canada; Ochsner Medical Center, New Orleans, Louisiana; Central Northern Adelaide Renal and Transplantation Service, Adelaide, South Australia, Australia; Formerly Merck & Co., Inc., Rahway, New Jersey; Merck & Co., Inc., Rahway, New Jersey; Merck & Co., Inc., Rahway, New Jersey; Merck & Co., Inc., Rahway, New Jersey

## Abstract

**Background:**

VGCV is approved for prophylaxis in adult KTRs at high risk for CMV disease (donor CMV-seropositive/recipient CMV-seronegative [D+/R-]); however, its use is limited by myelosuppression. LET is non-myelotoxic, does not require dose modification in renal impairment, and is approved for prophylaxis of CMV infection and disease in adult CMV-seropositive allogeneic hematopoietic stem cell transplant recipients. This randomized, double-blind, Phase 3 non-inferiority study (NCT03443869; MK-8228-002) evaluated CMV prophylaxis with LET vs. VGCV in adult CMV D+/R- KTRs.

**Methods:**

Adult CMV D+/R- KTRs were randomized 1:1 within 7 days post-kidney transplant (KT) to receive either LET 480 mg QD (PO/IV) with acyclovir (400 mg PO BID, adjusted for renal function), or VGCV (900 mg PO QD, adjusted for renal function), through Week 28 post-KT and followed up through Week 52 post-KT. Randomization was stratified by use of lymphocyte-depleting induction immunosuppression. The primary endpoint was the proportion of participants (pts) with CMV disease adjudicated by blinded committee through Week 52 post-KT (non-inferiority margin,10%).

**Results:**

A total of 601 pts were randomized; 589 received ≥ 1 dose of study medication (median age [range], 51 [18–82] years; male, 72%; Black/African American, 9%; deceased donor, 60%; received lymphocyte-depleting induction immunosuppression, 46%), of whom two had detectable CMV viral DNA on Day 1 and one was CMV R+ (full analysis set, 586 pts). The proportion of pts with CMV disease through Week 52 post-KT was 10.4% with LET vs. 11.8% with VGCV (stratum-adjusted treatment difference, -1.4% [95% CI -6.5, 3.8]; **Table 1**). Drug-related adverse events (AEs) were reported in 19.9% of pts with LET and 35.0% of pts with VGCV through Week 28 post-KT. The rate of discontinuations due to an AE was 4.1% in the LET arm and 13.5% in the VGCV arm (**Table 2**). The incidence of neutropenia (absolute neutrophil count < 1000/µL) during the treatment phase was lower with LET than with VGCV (4.1% vs. 19.5%; difference, -15.4% [95% CI -20.7, -10.5]).

**Figure:**
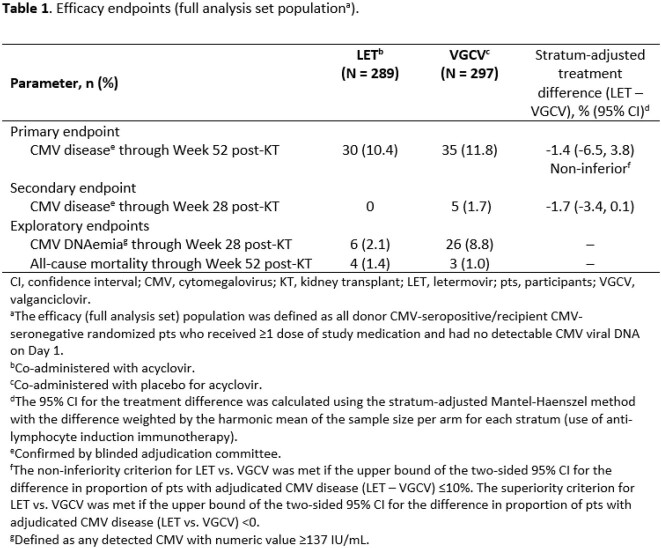

**Figure:**
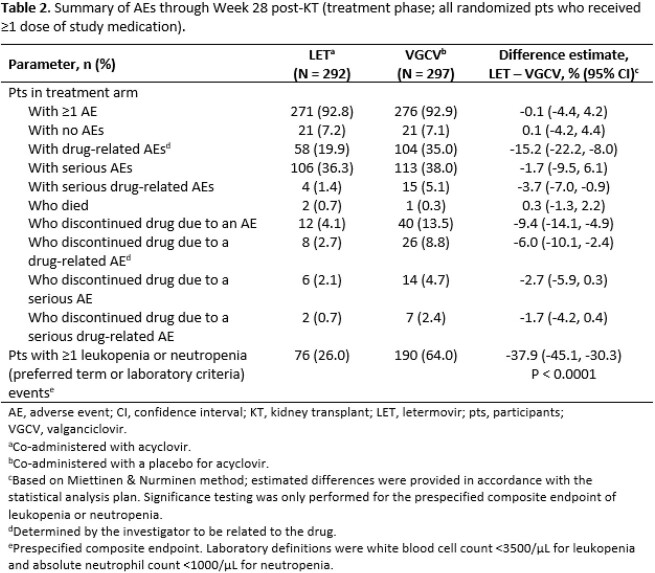

**Conclusion:**

The study met its primary endpoint: LET was non-inferior to VGCV in preventing CMV disease in high-risk (CMV D+/R-) KTRs through Week 52 post-KT, and led to a lower rate of myelotoxicity than VGCV.

**Disclosures:**

**Ajit P. Limaye, MD**, AiCuris: Advisor/Consultant|MedPace: Data Safety Monitoring Boards|Merck: Advisor/Consultant|Merck: Grant/Research Support|Moderna: Advisor/Consultant|Novartis: Data Safety Monitoring Boards|Sana: Advisor/Consultant **Klemens Budde, MD**, Abbvie: Grant/Research Support|Abbvie: Honoraria|Alexion: Grant/Research Support|Alexion: Honoraria|Astellas: Grant/Research Support|Astellas: Honoraria|Bristol Myers Squibb: Grant/Research Support|Bristol Myers Squibb: Honoraria|Chiesi: Grant/Research Support|Chiesi: Honoraria|CSL Behring: Grant/Research Support|CSL Behring: Honoraria|Fresenius: Grant/Research Support|Fresenius: Honoraria|Hansa: Grant/Research Support|Hansa: Honoraria|Hexal: Grant/Research Support|Hexal: Honoraria|Hookipa Biotech: Grant/Research Support|Hookipa Biotech: Honoraria|Merck Sharp & Dohme: Grant/Research Support|Merck Sharp & Dohme: Honoraria|Novartis: Grant/Research Support|Novartis: Honoraria|Otsuka: Grant/Research Support|Otsuka: Honoraria|Pfizer: Grant/Research Support|Pfizer: Honoraria|Roche: Grant/Research Support|Roche: Honoraria|Sandoz: Grant/Research Support|Sandoz: Honoraria|Shire: Grant/Research Support|Shire: Honoraria|Siemens: Grant/Research Support|Siemens: Honoraria|Takeda: Grant/Research Support|Takeda: Honoraria|Veloxis: Grant/Research Support|Veloxis: Honoraria|Vitaeris: Grant/Research Support|Vitaeris: Honoraria **Atul Humar, MD, MSc, FRCPC**, Merck: Advisor/Consultant|Merck: Grant/Research Support|Roche: Grant/Research Support|Takeda: Advisor/Consultant **Julia Garcia-Diaz, MD**, Astellas Pharma US, Inc.: Advisor/Consultant|Astellas Pharma US, Inc.: Speaker's Bureau **Robert P. Carroll, BM BCh (Oxon), FRACP, DM (Oxon)**, Bristol Myers Squibb: Grant/Research Support|HANSA Biopharma: Advisor/Consultant **Yoshihiko Murata, MD, PhD**, Former employee of Merck Sharp & Dohme LLC, a subsidiary of Merck & Co., Inc.,: Stocks/Bonds **Valerie L. Teal, MS**, Merck Sharp & Dohme LLC, a subsidiary of Merck & Co., Inc.,: Current Employee|Merck Sharp & Dohme LLC, a subsidiary of Merck & Co., Inc.,: Stocks/Bonds **Christopher L. Gilbert, n/a**, Current employee of Merck Sharp & Dohme LLC, a subsidiary of Merck & Co., Inc.,: Stocks/Bonds **Barbara A. Haber, MD**, Current employee of Merck Sharp & Dohme LLC, a subsidiary of Merck & Co.,: Stocks/Bonds.

